# Reduced-order modelling of parameter-dependent, linear and nonlinear dynamic partial differential equation models

**DOI:** 10.1098/rspa.2016.0809

**Published:** 2017-04-26

**Authors:** A. A. Shah, W. W. Xing, V. Triantafyllidis

**Affiliations:** School of Engineering, University of Warwick, Coventry CV4 7AL, UK

**Keywords:** parameter-dependent partial differential equations, proper orthogonal decomposition, Gaussian process model, manifold learning, nonlinear systems

## Abstract

In this paper, we develop reduced-order models for dynamic, parameter-dependent, linear and nonlinear partial differential equations using proper orthogonal decomposition (POD). The main challenges are to accurately and efficiently approximate the POD bases for new parameter values and, in the case of nonlinear problems, to efficiently handle the nonlinear terms. We use a Bayesian nonlinear regression approach to learn the snapshots of the solutions and the nonlinearities for new parameter values. Computational efficiency is ensured by using manifold learning to perform the emulation in a low-dimensional space. The accuracy of the method is demonstrated on a linear and a nonlinear example, with comparisons with a global basis approach.

## 1. Introduction

Computational modelling is an indispensable tool for analysis, design, optimization and control. For applications that require a high number of model evaluations at different inputs (e.g. uncertainty analysis and inverse parameter estimation) the computational expense of a computer model is often prohibitive. In such cases, the original computer model can be replaced with a *surrogate model* (or *emulator*) [1]. The simplest approach to surrogate modelling consists of simplifying the mathematical model or numerical formulation, e.g. by assuming spatial homogeneity or using coarse grids.

The other two main approaches are based on: (i) supervised machine learning methods to learn the model input–output relationship (so-called data-driven models) or (ii) Galerkin projection schemes, to yield *reduced-order models* (ROMs). The projecting basis in ROMs can be obtained through balanced truncation, Krylov subspaces or proper orthogonal decomposition (POD) (for a recent survey we refer the reader to [2]). For partial differential equation (PDE) models, the Galerkin projection can be performed on the original equations (strong or a weak form) or on the spatially discretized system. The final form is an algebraic system for steady problems or an ordinary differential equation (ODE) system in time for dynamical problems.

The most widely used technique for PDE systems is POD [3–5], in which the approximating subspace is obtained from solutions (called *snapshots*) generated by the discretized full-order model (FOM) at selected time instances. Application of POD to dynamic, nonlinear parametrized PDEs presents a number of challenges: (i) constructing a basis that is valid across parameter space; (ii) dealing with high-dimensional parameter spaces; (iii) using data parsimoniously; and (iv) efficiently computing the reduced-order system matrices and reduced-order nonlinearities in the state variable during use of the surrogate, i.e. the so-called *online* phase (we may also mention the development of stable POD schemes to overcome instabilities in the original formulations).

There are several approaches to incorporating parametric dependence: (i) to use a global basis (meaning across parameter space); (ii) interpolation of local bases (meaning for particular parameter values); and (iii) interpolation of local system matrices. For linear time-invariant systems, the system matrices often take the form of affine combinations of constant matrices with parameter-dependent coefficients. In such cases, the reduced-order system is quickly and easily assembled for a new parameter value [6,7]. Affine forms can also be realized by using a Taylor series expansion [8] or an empirical interpolation strategy [9]. Global basis methods extract a single basis from multiple local snapshot matrices [6,10,11]. Obvious drawbacks are the violation of POD optimality and the growth in the size of the global matrix with the number of samples. There are, however, efficient sampling strategies for constructing global bases, such as the greedy approach of [6] or by using a local sensitivity analysis [12].

An alternative approach is interpolation of local bases or local reduced-model matrices. Lieu *et al.* [13] used the principal angles between two POD bases, pertaining to different Mach numbers, to linearly interpolate a local basis for intermediate Mach numbers in a linearized fluid-structure ROM. This method is restricted to single-parameter systems and small parameter changes. Amsallem & Farhat [14] considered local bases as members of a Grassmann manifold, the set of all subspaces (of a chosen low dimension) of the state space. The local bases are mapped to a tangent space of the Grassman manifold using a logarithmic map and Lagrange interpolation is performed in the tangent space. An inverse exponential map provides the required local bases.

Interpolation methods can also be used to approximate the reduced-order system matrices, in order to circumvent the problem of computing these matrices for each new parameter value. Degroote *et al.* [11] proposed two methods: element-wise direct spline interpolation of the reduced-order matrices or spline interpolation of the matrices in a tangent space of a Riemannian manifold on which the matrices are assumed to lie (a similar method was proposed in [15]). When a global basis is not used to build the ROM, a straightforward interpolation is not possible because the reduced-dimensional coordinates do not (in general) have the same physical meaning from one local basis to another. Thus, a congruency transformation to a common basis is required before direct interpolation [16] or interpolation in a tangent space [17].

Lieberman *et al.* [18] used a greedy algorithm to construct projections for both the state variable and the parameters simultaneously, minimizing a measure of the error between the ROM and FOM outputs at each iteration (different error measures were considered in [19]). Hay *et al.* [20] used sensitivities (derivatives) of the POD basis with respect to (w.r.t.) the parameters either to linearly extrapolate the POD basis for a new parameter value or to expand the POD basis by augmenting it with the corresponding sensitivities. The growth in the basis dimension with the number of parameters is a limitation of this approach.

The computational cost of evaluating a strong (high-order polynomial or non-polynomial) nonlinearity in the state variable in a ROM depends on the dimension of the original state space. Linearization methods [21,22] are only applicable to weak nonlinearities or confined regions of state space. Moreover, the computational cost grows exponentially with the order of the approximating expansion. Recently, a number of *hyper-reduction* methods have been developed to overcome the limitations of linearization approaches (see also the tensorial POD approach recently developed in [23]). An early method was developed by Astrid *et al.* [24], based on selecting a subset of the FOM equations corresponding to heuristically chosen spatial grid points, followed by a Galerkin projection of the resulting reduced system onto the POD basis.

The empirical interpolation method interpolates the nonlinear function at selected spatial locations using an empirically derived basis, and is applied directly to the governing PDE [7], while the *discrete empirical interpolation method* (DEIM) is applicable to general ODE or algebraic systems arising from a spatial discretization [25]. Both methods construct a subspace for the approximation of the nonlinear term and use a greedy algorithm to select interpolation points. An extension of the DEIM [26] generates several local subspaces via clustering and uses classification in the online phase to select one of the subspaces. These approaches can also be used for approximating (vectorized) non-affine system matrices [2]. The Gauss–Newton with approximated tensors method operates at the fully discrete level in space and time, and is based on satisfying consistency and discrete-optimality conditions by solving a residual-minimization problem [27]. This leads to a Petrov–Galerkin (rather than Galerkin) problem with a test basis that depends on the residual derivatives w.r.t. the state variable.

In this paper, we introduce an extension of POD for dynamic, parametrized, linear and nonlinear PDEs. The method we develop involves a computationally efficient approximation of the POD basis and the nonlinearity for new parameter values. It can be used in conjunction with many of the methods described above, e.g. greedy sampling and methods for approximating non-affine system matrices. In order to avoid inconsistencies and to reduce the loss of information in the construction of new bases, we take the approach of approximating the snapshots rather than the bases or system matrices directly. The snapshots, however, lie in high-dimensional spaces so that direct approximations are computationally unfeasible. We overcome this issue by using manifold learning techniques [28] to map the snapshots to a low-dimensional feature space. We then use Gaussian process emulation (GPE) to infer values of the mapped snapshots for new parameter values, followed by an inverse map to obtain the snapshots in physical space. For nonlinear problems, we extend DEIM by using the same emulation approach to approximate snapshots of the nonlinearity at desired locations in parameter space.

In the next section, we outline the procedures for generating ROMs and POD bases. We provide brief details of the DEIM and explain the issues associated with parametrized and/or nonlinear problems. In §3, we present the snapshot emulation strategy and summarize our approach to linear and nonlinear parametrized problems. In §4, we present one linear and one nonlinear example, comparing the results with a global basis approach.

## 2. Reduced-order models for parametrized dynamic partial differential equations using proper orthogonal decomposition

### (a) Problem definition and Galerkin projection

Let ***x***=(*x*_1_,…,*x*_*L*_) denote a point in a bounded, regular domain $D\subset R^{L}$ (*L*=1,2,3), let *t*∈[0,*T*] denote time and let $\xi \in X\subset R^{l}$ denote a vector of parameters. For the purposes of illustration, consider a parametrized, parabolic PDE for a dependent variable *u*(***x***,*t*;***ξ***):

2.1 $$\begin{array}{rl} \partial_{t}u+L(\xi )u+N(\xi )u & \;=g(\text{x};\xi )\;(\text{x},t)\in D\times (0,T]\\ \text{and}\;u(\text{x},0;\xi ) & \;=u_{0}(\text{x};\xi )\;\text{x}\in D, \end{array}\}$$

augmented by linear boundary conditions. Here, L(ξ) and N(ξ) are parameter-dependent linear and nonlinear partial differential operators, respectively. The dependence on the parameters can be through the operators, the source term *g*(***x***;***ξ***) or the initial/boundary conditions.

Let H be a separable Hilbert space with inner product ${\left(\cdot ,\cdot \right)}_{H}$ and induced norm $∥\cdot ]{|}_{H}$, e.g. $L^{2}(D)$, the space of square integrable equivalence classes of functions with inner product ${\left(v,v^{\prime }\right)}_{L^{2}(D)}=\int_{D}v(\text{x})v^{\prime }(\text{x})\;d\text{x}$. Henceforth, we drop the subscript in the notation for the inner product and norm in $L^{2}(D)$. It is assumed that, for each ***ξ***, $u\in L^{2}(0,T;H)$, i.e. *t*↦*u*(⋅,*t*;***ξ***) is a Lebesgue measurable map from (0,*T*) to H with finite norm $∥u{∥}_{L^{2}(0,T;H)}:=\int_{0}^{T}∥u(\cdot ,t;\xi ){∥}_{H}\;dt$. Then u(⋅,t;ξ)∈H for each *t*∈(0,*T*). A spatial discretization of (2.1) leads to a system of ODEs:

2.2 $$\dot{\text{u}\;}\;\;(t;\xi )=\text{A}(\xi )\text{u}(t;\xi )+\text{f}(\text{u}(t;\xi );\xi )\;\mathrm{and}\;\text{u}(0;\xi )={\text{u}}_{0}(\xi )$$

for a discrete state variable ***u***(*t*;***ξ***)=(*u*_1_(*t*;***ξ***),…,*u*_*d*_(*t*;***ξ***))^T^, which we call the solution vector. Here *d* is the number of degrees of freedom, e.g. the number of grid points in a finite-difference (FD) approximation, the number of cells in a cell-centred finite-volume (FV) approximation or the number of nodes (basis functions) in a finite-element (FE) approximation. The matrix $\text{A}(\xi )\in R^{d\times d}$ arises from the linear term L(ξ)u and $\text{f}(\text{u}(t;\xi );\xi )\in R^{d}$ arises from a combination of N(ξ)u, *g*(***x***;***ξ***) and possibly the boundary conditions. The latter is nonlinear for N(ξ)u≢0.

The precise relationship between ***u***(*t*;***ξ***) and *u*(***x***,*t*;***ξ***), the forms of **A**(***ξ***) and ***f***(***u***;***ξ***), and the incorporation of boundary conditions depend on the method used. For an FD approximation, problem (2.1) is solved directly and the boundary conditions are incorporated in ***f***(***u***;***ξ***). In an FE approximation a weak form is solved with test functions in H or a dense subspace V of H, with boundary conditions incorporated in ***f*** and/or the definition of H. The form of **A**(***ξ***) is determined by the dependence of L(ξ) on ***ξ***. The simplest case is an affine form: $\text{A}(\xi )=\sum_{i}c_{i}(\xi ){\text{A}}_{i}$, where the functions *c*_*i*_(***ξ***) are known and the matrices **A**_*i*_ are constant.

For FD, FV and nodal-basis FE discretizations, the coefficients *u*_*i*_(*t*;***ξ***) of ***u***(*t*;***ξ***) correspond to the values of *u*(***x***,*t*;***ξ***) at locations ${\text{x}}_{i}\in \bar{D}$, *i*=1,…,*d*, i.e. *u*_*i*_(*t*;***ξ***)=*u*(***x***_*i*_,*t*;***ξ***). We will assume this to be the case throughout. A numerical solution of (2.2) yields the solution vector ***u***_*i*_(***ξ***):=***u***(*t*_*i*_;***ξ***) at times ${\left\{t_{i}\right\}}_{i=1}^{m}$. Each of the discrete solutions ${\text{u}}_{i}(\xi )\in R^{d}$ is referred to as a *snapshot*.

For a fixed input ξ∈X, a Galerkin projection approximates the problem (2.2) in a proper (low-dimensional) subspace S(ξ) of $R^{d}$. Let ${\text{v}}_{j}(\xi )\in R^{d}$, *j*=1,…,*r*, be an orthonormal basis for S(ξ) (dim(S(ξ))=r≪d), where the notation makes explicit the dependence on the input. We seek an approximation u(t;ξ)∈S of ***u*** in the space span(***v***_1_(***ξ***),…,***v***_*r*_(***ξ***))

2.3 $$\text{u}(t;\xi )=\sum_{j=1}^{r}a_{j}(\text{t};\xi ){\text{v}}_{j}(\xi )={\text{V}}_{r}(\xi )\text{a}(t;\xi ),$$

where ***a***=(*a*_1_(*t*;***ξ***),…,*a*_*r*_(*t*;***ξ***))^T^ and **V**_*r*_(***ξ***)=[***v***_1_(***ξ***)…***v***_*r*_(***ξ***)]. The Galerkin projection of equations (2.2) onto the basis vectors ***v***_*i*_(***ξ***), *i*=1,…,*r*, yields (replacing ***u*** with **u**)

2.4 $$\dot{\text{a}\;}\;\;(t;\xi )={\text{A}}_{r}(\xi )\text{a}(t;\xi )+{\text{f}}_{r}\left(\text{a}(t;\xi );\xi \right)\;\mathrm{and}\;\text{a}(0;\xi )={\text{V}}_{r}{\left(\xi \right)}^{T}{\text{u}}_{0}(\xi ),$$

where **A**_*r*_(***ξ***):=**V**_*r*_(***ξ***)^T^**A**(***ξ***)**V**_*r*_(***ξ***) and **f**_*r*_(***a***(*t*;***ξ***);***ξ***):=**V**_*r*_(***ξ***)^T^***f***(**V**_*r*_(***ξ***)***a***(*t*;***ξ***);***ξ***). Equations (2.4) represent a system of *r* ODEs in time for the coefficients *a*_*i*_(*t*;***ξ***). The basic goal of POD (outlined below) is the construction of a basis ${\left\{{\text{v}}_{j}(\xi )\right\}}_{j=1}^{r}$ using the snapshots ${\left\{{\text{u}}_{i}(\xi )\right\}}_{i=1}^{m}$.

### (b) Proper orthogonal decomposition

POD is presented in a number of ways (e.g. error minimization, ‘variance’ maximization) in the literature and often under different names. In this section, we provide a brief description of the motivation and practical (discrete) implementation. A complete summary of the underlying theory, alternative approaches, the links between the various interpretations and the optimality of the chosen basis can be found in appendix A.

For a fixed ξ∈X, POD extracts an ‘optimal’ basis for a field *u*(***x***,*t*;***ξ***), (x,t)∈D×[0,T], given an ensemble of ‘snapshots’ ${\left\{u(\text{x};t_{j},\xi )\right\}}_{j=1}^{m}$, x∈D. These are continuous equivalents of the discrete snapshots ***u***_*j*_(***ξ***). *u*(***x***,*t*;***ξ***) can be regarded as a realization of a stationary (w.r.t. *t*) random field indexed by (***x***,*t*) [3,4,29]. Applying Karhunen–Loèéve (KL) theory [30] *for a fixed* *t* yields $u(\text{x},t;\xi )=\lim_{M\to \infty }\sum_{i=1}^{M}a_{i}(t;\xi )v_{i}(\text{x};\xi )$. The *v*_*i*_(***x***;***ξ***) form an $L^{2}(D)$ orthonormal basis and are the eigenfunctions (equations (.1) in appendix A) of an integral operator C with the kernel given by the *spatial autocovariance function* *C*(***x***,***x***′;***ξ***), $\text{x},{\text{x}}^{\prime }\in D$.

In practice, we must work within a finite-dimensional setting. Defining **U**(***ξ***):=[***u***_1_(***ξ***)…***u***_*m*_(***ξ***)], the *spatial variance–covariance matrix* is given by $\text{C}(\xi )=\text{U}(\xi )\text{U}{\left(\xi \right)}^{T}\approx E[\text{u}(t;\xi )\text{u}{\left(t;\xi \right)}^{T}]$. The continuous eigenvalue problem for C can be approximated numerically (non-uniquely) by a principal component analysis (PCA): **C**(***ξ***)***v***_*i*_(***ξ***)=*λ*_*i*_(***ξ***)***v***_*i*_(***ξ***) for eigenvectors ${\text{v}}_{i}(\xi )\in R^{d}$ and eigenvalues *λ*_*i*_(***ξ***)>0, *i*=1,…,*d*, arranged in decreasing order. The first *r* of these vectors define the space S(ξ) of §2a. In certain cases, it may be computationally convenient to use variants of POD/PCA to determine the ***v***_*i*_(***ξ***). In appendix A, we provide details of the *method of snapshots* and *singular value decomposition* (SVD), the latter of which we use in practice.

## 3. Basis emulation and discrete empirical interpolation method extension

For each input/parameter ***ξ***, the snapshot matrix **U**(***ξ***) is obtained from the FOM and the basis **V**_*r*_(***ξ***) is constructed according to §2b. To perform an analysis w.r.t. the inputs, this procedure is computationally prohibitive. A global basis across the parameter space of interest [10] can be constructed by computing a set of snapshot matrices **U**(***ξ***_*j*_) for $\xi_{j}\in X$, *j*=1,…,*n*. The ***v***_*i*_(***ξ***) are extracted from a global snapshot matrix $[\text{U}(\xi_{1}),\ldots ,\text{U}(\xi_{n})]\in R^{d\times nm}$ (usually after an SVD to avoid rank deficiency).

The global basis method uses information only from the ‘truth approximation’, i.e. the FOM. The optimality of the POD method, on the other hand, is violated since the snapshots used to derive the basis do not pertain to the parameter value of interest (the particular dynamical system under consideration) during the online phase. Furthermore, the range of validity of the global basis could be limited for complex mappings between the parameters and the outputs [13]. Interpolation methods (and the method we propose) violate the truth approximation in the sense that the snapshots or quantities derived therein are not obtained from the original model. In contrast to the global basis, however, these methods attempt to construct more accurate ROMs during the online phase. The main limitation is the accuracy of the interpolation or emulation, which depends on the data available and on the method itself. Moreover, it may not be possible to obtain sharp error bounds using such methods (in cases where the underlying PDE problem is amenable to a rigorous analysis).

Another problem associated with the standard POD–Galerkin approach is that the computational efficiency is compromised when $\text{f}(\cdot ;\xi )\in R^{d}$ is a strong nonlinearity, since the evaluation of **f**_*r*_ in equation (2.4) has a computational complexity that depends on *d* [31]. The DEIM [25] seeks a set of vectors ${\text{w}}_{i}(\xi )\in R^{d}$, *i*=1,…,*d*, such that the subspace $\text{span}({\text{w}}_{1}(\xi ),\ldots ,{\text{w}}_{s}(\xi ))\subset R^{d}$ for some *s*≪*d* well approximates ***f***(***u***(*t*;***ξ***);***ξ***) for an arbitrary *t*. That is, an approximation ***f***(***u***(*t*;***ξ***);***ξ***)≈**W**(***ξ***)***h***(*t*;***ξ***), where **W**(***ξ***)=[***w***_1_(***ξ***)…***w***_*s*_(***ξ***)] and $\text{h}(t;\xi )\in R^{s}$. The basis ${\left\{{\text{w}}_{i}(\xi )\right\}}_{i=1}^{d}$ is constructed from snapshots of the nonlinearity ${\left\{{\text{f}}_{i}(\xi )\right\}}_{i=1}^{m}$, where ***f***_*i*_(***ξ***)=***f***(***u***_*i*_(***ξ***);***ξ***), from which we form the matrix **F**(***ξ***)=[***f***_1_(***ξ***)…***f***_*m*_(***ξ***)]. A PCA on **F**(***ξ***)**F**(***ξ***)^T^ or SVD of **F**(***ξ***) yields the ${\left\{{\text{w}}_{i}(\xi )\right\}}_{i=1}^{d}$, arranged such that the corresponding eigenvalues decay with *i*.

Since the system ***f***(***u***(*t*;***ξ***);***ξ***)=**W**(***ξ***)***h***(*t*;***ξ***) is overdetermined in ***h***(*t*;***ξ***), the DEIM selects *s* of the *d* equations to obtain an ‘optimal’ solution. Let us introduce the matrix $\text{P}=[{\text{e}}_{p_{1}}\ldots {\text{e}}_{p_{s}}]\in R^{d\times s}$, where ***e***_*p*_*i*__ is the standard Euclidean basis vector in $R^{d}$ with non-zero entry located at the *p*_*i*_th coordinate. Assuming **P**^T^**W**(***ξ***) is non-singular, we obtain

3.1 $$\begin{array}{rl} {\text{f}}_{r}(\text{a}(t;\xi );\xi )\approx {\text{V}}_{r}{\left(\xi \right)}^{T}\text{W}(\xi )\text{h}(t;\xi ) & \;={\text{V}}_{r}{\left(\xi \right)}^{T}\text{W}(\xi ){\left({\text{P}}^{T}\text{W}(\xi )\right)}^{-1}{\text{P}}^{T}\text{f}(\text{u}(t;\xi );\xi )\\ & \;={\text{V}}_{r}{\left(\xi \right)}^{T}\text{W}(\xi ){\left({\text{P}}^{T}\text{W}(\xi )\right)}^{-1}\text{f}({\text{P}}^{T}\text{u}(t;\xi );\xi ), \end{array}$$

assuming that the function ***f***(⋅;***ξ***) acts pointwise. The indices *p*_*i*_∈{1,2,…,*d*}, *i*=1,…,*s* are specified by a greedy algorithm [25] that satisfies the following error bound (for a given *s*):

3.2 $$∥\text{f}-\hat{\text{f}\;}\;\;∥\leq ∥{\left({\text{P}}^{T}\text{W}(\xi )\right)}^{-1}∥\;∥(\text{I}-\text{W}(\xi )\text{W}{\left(\xi \right)}^{T})\text{f}∥,$$

where ∥⋅∥ is the standard Euclidean norm and $\hat{\text{f}\;}\;\;:=\text{W}(\xi ){\left({\text{P}}^{T}\text{W}(\xi )\right)}^{-1}{\text{P}}^{T}\text{f}$ is the DEIM approximation of ***f***. This estimate is valid for a given *t* (considering ***f*** as a function of *t*) by virtue of the second factor on the r.h.s., which is the error in the best 2-norm approximation of ***f*** in range(**W**(***ξ***)).

In this paper, we introduce a systematic and rigorous method to approximate the local basis and the nonlinearity by first approximating the snapshots ${\left\{{\text{u}}_{i}(\xi )\right\}}_{i=1}^{m}$ and ${\left\{{\text{f}}_{i}(\xi )\right\}}_{i=1}^{m}$ for an arbitrary input ***ξ*** using Bayesian nonlinear regression. These snapshots lie in very high-dimensional spaces and thus we use a recently developed method that exploits manifold learning to yield a computationally feasible Gaussian process (GP) model. Below we describe the components of this emulation method and subsequently explain how it can be used for a POD analysis of parameterized, dynamic problems.

### (a) Formulation and solution of the learning problem

For an arbitrary input ***ξ***, consider the mapping $\eta :X\to O\subset R^{md}$ defined below:

3.3 $$\text{y}=\eta (\xi )={\left({\text{u}}_{1}{\left(\xi \right)}^{T},\ldots ,{\text{u}}_{m}{\left(\xi \right)}^{T}\right)}^{T}\in R^{md},$$

i.e. a vectorial rearrangement of snapshots ${\left\{{\text{u}}_{i}(\xi )\right\}}_{i=1}^{m}$ for the given value of ***ξ***. We can define a similar map ***y***^*f*^=***η***^*f*^(***ξ***) for snapshots of the nonlinearity ${\left\{{\text{f}}_{i}(\xi )\right\}}_{i=1}^{m}$. The emulation procedure mirrors that described below for the snapshots ${\left\{{\text{u}}_{i}(\xi )\right\}}_{i=1}^{m}$.

We aim to approximate the mapping ***η***(⋅) given *training points* ${\text{y}}_{j}=\eta (\xi_{j})\in O$ (in a high-dimensional space) for *design points* $\xi_{j}\in X$, *j*=1,…,*n*. One of the main methods for dealing with such high-dimensional outputs is to define approximate outputs in a *q*-dimensional subset $O_{q}\subset O$ (*q*≪*md*) using PCA and independently emulate the *q* coefficients of the points in $O_{q}$ for new values of ***ξ*** [32]. Shah and co-workers [33,34] extended the latter method by replacing PCA with manifold learning methods, making it applicable to a broader class of output spaces O. In this paper, we employ the method of [33,34] with kernel PCA (kPCA), which is outlined in appendix B, together with an approximation of the inverse map. kPCA [35] defines a map $\phi_{q}:O\to F_{q}$, where $F_{q}$ is a *q*-dimensional feature space. The coordinates *z*_*i*_(***y***) of points ***ϕ***_*q*_(***y***) in $F_{q}$ define composite maps from the input space X to R, i.e. ${\mathbb{Z}}_{i}(\xi ):=z_{i}(\eta (\xi ))$, *i*=1,…,*q*. We place independent GP priors over these maps, justified by the properties of kPCA.

The approximation of η:X→O given the training points ${\left\{{\text{y}}_{j}\right\}}_{j=1}^{n}$ is then substituted for independent approximations of the coefficients ${\text{ℤ}}_{i}(\xi )$, *i*=1,…,*q*, given training data ${\left\{{\text{ℤ}}_{i}(\xi_{j})=z_{i}(\eta (\xi_{j}))\right\}}_{j=1}^{n}$, which is obtained from equation (B 2) in appendix B. The value of ${\text{ℤ}}_{i}(\xi )$ for a new input ***ξ*** is inferred from scalar GPE (outlined in appendix C) as the mean of a posterior distribution. Given ${\left\{{\text{ℤ}}_{i}(\xi )\right\}}_{i=1}^{q}$, an approximation of the inverse $\phi_{q}^{-1}:F_{q}\to O$ yields an approximation of y=η(ξ)∈O, from which we can obtain ${\left\{{\text{u}}_{i}(\xi )\right\}}_{i=1}^{m}$ using definition (3.3). GPE is exact at the training points if there are no (spurious) errors in the training data. In the present case, an error is introduced in the pre-image map so that the training snapshots will not be recovered exactly. This error, however, is negligible (§4). We note that the size of *md* is not a limitation for the manifold learning methods employed in this paper, in which the eigenvalue problems are primarily dependent on the number of training points *n*.

### (b) Main algorithm

Once the snapshots ${\left\{{\text{u}}_{i}(\xi )\right\}}_{i=1}^{m}$ (and ${\left\{{\text{f}}_{i}(\xi )\right\}}_{i=1}^{m}$ for nonlinear problems) are obtained using the procedure outlined in §2b for a new input ***ξ***, POD can be performed in the usual manner (with the extended DEIM for nonlinear problems). The entire procedure is outlined in algorithm 1. We mention that kPCA can be replaced with other manifold learning methods, e.g. diffusion maps or Isomap [33,34]. We introduce the terminology ‘kGPE-POD’ to denote the method of algorithm 1 without the extended DEIM (i.e. steps 1a–7a alone). Similarly, we use the terminology ‘kGPE-POD-DEIM’ to denote the method of algorithm 1 with the extended DEIM (steps 1a–7a *and* steps 1b–7b together).


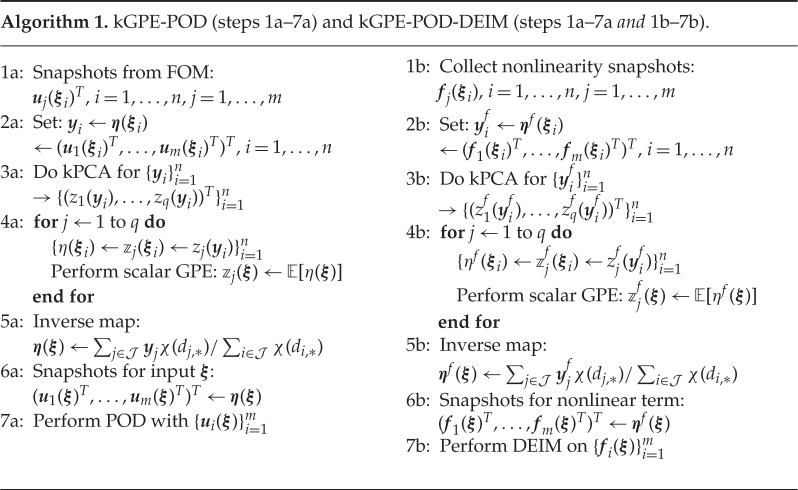


## 4. Results and discussion

### (a) Two-dimensional contaminant transport

We consider the transport of a contaminant governed by a convection–diffusion equation. This model can be used, for example, for real-time prediction or for quantifying uncertainty in the concentration to support decision-making [11]. The problem is specified as follows:

4.1 $$\begin{array}{r} \partial_{t}u+\text{q}\cdot \nabla u-\mu \nabla^{2}u=0\;\text{x}=(x_{1},x_{2})\in D:=[0,1]\times [0,1]\\ \mathrm{and}\;u=0\;\text{x}\in \partial D,\;u(\text{x},t)=u_{0}\;t=0 \end{array}\},$$

where *u*(***x***,*t*;***ξ***) denotes the contaminant concentration (mol m^−3^), ***q*** is the fluid velocity (m s^−1^) and *μ* is the contaminant diffusion coefficient (m^2^ s^−1^). The input ***ξ*** is defined below. The initial concentration is given by $u_{0}(\text{x})={\left(2\pi k_{0}\right)}^{-1/2}\sum_{i=1}^{3}{\text{k}}_{i}\exp(-k_{0}{\left(\text{x}-{\text{x}}_{i}\right)}^{T}(\text{x}-{\text{x}}_{i})/2)$, where ***x***_1_=(0.2,0.2)^T^, ***x***_2_=(0.2,0.8)^T^, ***x***_3_=(0.8,0.8)^T^, *k*_0_=0.01, *k*_1_=1, *k*_2_=2 and *k*_3_=3. The magnitude of the velocity field is inversely proportional to the distance from $\text{x}={\left({\hat{x}}_{1},{\hat{x}}_{2}\right)}^{T}$,

4.2 $$\text{q}(\text{x})=\frac{a_{1}(x_{1}-{\hat{x}}_{1}){\text{e}}_{1}+a_{2}(x_{2}-{\hat{x}}_{2}){\text{e}}_{2}}{{\left(x_{1}-{\hat{x}}_{1}\right)}^{2}+{\left(x_{2}-{\hat{x}}_{2}\right)}^{2}},$$

where ***e***_1_ and ***e***_2_ are unit vectors in the *x*_1_- and *x*_2_-directions, respectively, and $a_{i}\in R$. To avoid the singularity at $\text{x}={\left({\hat{x}}_{1},{\hat{x}}_{2}\right)}^{T}$, the norm of velocity is set to zero at this location. We also set *a*_1_=*a*_2_=1 and *μ*=1, and consider variations in the input $\xi ={\left({\hat{x}}_{1},{\hat{x}}_{2}\right)}^{T}\in X:=[0,1]\times [0,1]$.

The problem was discretized in space using a cell-centred FV method with *d*=2500 square cells (control volumes). Central differencing was used for the diffusive term and a first-order upwind scheme for the convective term, defining the velocity values on a staggered grid. A fully implicit Euler method was used to solve the resulting semi-discrete linear problem with 100 equal time steps in *t*∈[0,*T*], *T*=0.2 s. A total of 500 inputs $\xi_{j}\in X$, *j*=1,…,500, were generated using a Sobol sequence [36]. For each input, the FOM was solved to yield solution vectors (snapshots) ${\text{u}}_{i}(\xi_{j})\in R^{d}$, *i*=1,…,100, *j*=1,…,500. The data points (vectorized snapshots) ***y***_*j*_=***η***(***ξ***_*j*_), *j*=1,…,500, were obtained using equation (3.3). Referring to appendix A, we set $H=L^{2}(D)$ to define the POD basis and optimality. Of the 500 data points, *n*_*t*_=300 were reserved for testing. Training points were selected from the remaining 200 data points (*n*≤200).

A Gaussian kernel was used for kPCA. The free parameter *s*^2^ was taken to be the average square distance between observations in the original space [37]: $s^{2}=n^{-2}\sum_{i,j=1}^{n}∥{\text{y}}_{i}-{\text{y}}_{j}{∥}^{2}$. Polynomial, multi-quadratic and sigmoid kernels were also tested. The best performance was achieved with the sigmoid and Gaussian kernels. For the inverse mapping, *N*_*n*_=*n* was used (i.e. all training points). For the GPE, we used a squared exponential covariance function and a zero mean function (after centring). The hyperparameters were found using a maximum-likelihood estimate (MLE) (gradient descent). Errors in the predictions of the vectorized snapshots ***y***_*j*_ were measured using a normalized error: $\epsilon =∥{\text{y}}_{j}^{p}-{\text{y}}_{j}∥/∥{\text{y}}_{j}∥$, where ${\text{y}}_{j}^{p}$ denotes the prediction of the test point ***y***_*j*_=***η***(***ξ***_*j*_), *j*=1,…,*n*_*t*_, using steps 1a–6a of algorithm 1. Errors in the predictions using kGPE-POD/kGPE-POD-DEIM at ***ξ***_*j*_ were measured using a relative error *ϵ*_*r*_,

4.3 $$\epsilon_{r}=\frac{1}{m}\sum_{i=1}^{m}\frac{∥{\text{u}}_{i}^{p}(\xi_{j})-{\text{u}}_{i}(\xi_{j})∥}{∥{\text{u}}_{i}(\xi_{j})∥},$$

where ${\text{u}}_{i}^{p}(\xi_{j})$ is the prediction (steps 1a–7a in algorithm 1) of the test point (snapshot) ***u***_*i*_(***ξ***_*j*_).

**Figure 1.**
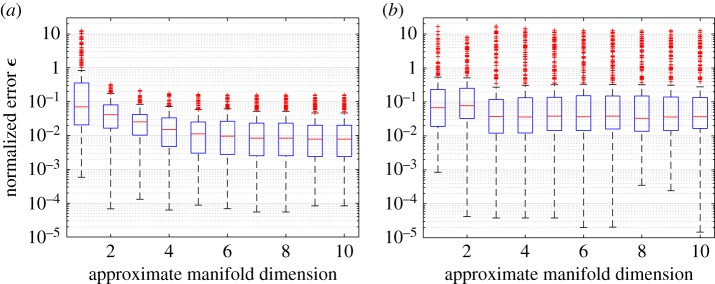
Tukey box plots of *ϵ* with increasing *q* for the contaminant transport model (*n*_*t*_=300, *n*=80 and *m*=10): (*a*) kPCA and (*b*) Isomap. (Online version in colour.)

We first examine the normalized errors *ϵ* in the predictions of the test data points ***y***_*j*_=***η***(***ξ***_*j*_), *j*=1,…,*n*_*t*_. Using *m*=10 of the snapshots (selecting every 10), figure 1 shows Tukey box plots of *ϵ* for the *n*_*t*_=300 test cases as the manifold dimension *q* is increased, using *n*=80 training points. Outliers are plotted individually using a ‘+’ symbol. We note that when predicting the training set in this case using *q*=10 the maximum value of *ϵ* was around 10^−11^, while the median was around 10^−12^. As a comparison we also include the result for Isomap (replacing kPCA in algorithm 1). The best results were obtained with kPCA, for which the errors converge after *q*=6 dimensions (negligible further decrease). Diffusion maps were also tested and gave results similar to kPCA. The same pattern was observed at *n*=40, 120 and 200 training points and also for all values of *m* up to 100. Based on the results, the approximating manifold dimension was set to *q*=10 for all values of *n* and *m* (using kPCA).

**Figure 2.**
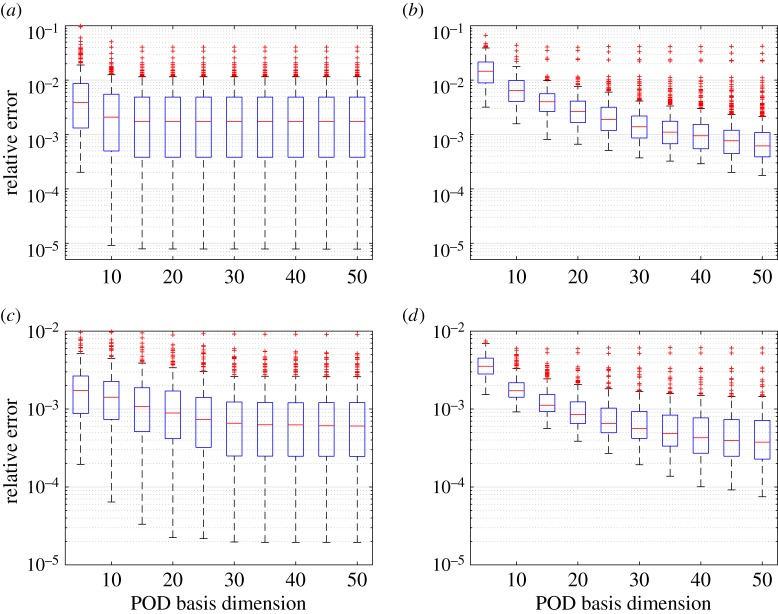
Tukey box plots of *ϵ*_*r*_ with increasing *r* for the contaminant transport model (*n*_*t*_=300 and *n*=80). (*a*) kGPE-POD with *m*=10, (*b*) global basis with *m*=10, (*c*) kGPE-POD with *m*=100 and (*d*) global basis with *m*=100. (Online version in colour.)

Figure 2 compares kGPE-POD with a global basis method for increasing POD dimension *r*. In the global basis method, the snapshot matrices constituting the global snapshot matrix corresponded to the *n*=80 training points used for kGPE-POD. An SVD was performed on the global matrix before extracting the POD basis. For *n*=40, the results were similar to the results depicted in figure 2, with a slight decrease in accuracy for both methods. Using *m*=10 snapshots, the decrease in the relative errors *ϵ*_*r*_ in kGPE-POD is negligible for *r*>15, while the global basis method continues to improve beyond *r*=50. In principle, kGPE-POD uses the correct bases for the test parameter values. It is possible, therefore, that kGPE-POD would approach the true result for a smaller value of *r* than the global basis approach, which uses a single basis extracted from snapshots that do not pertain to the test parameter values.

**Figure 3.**
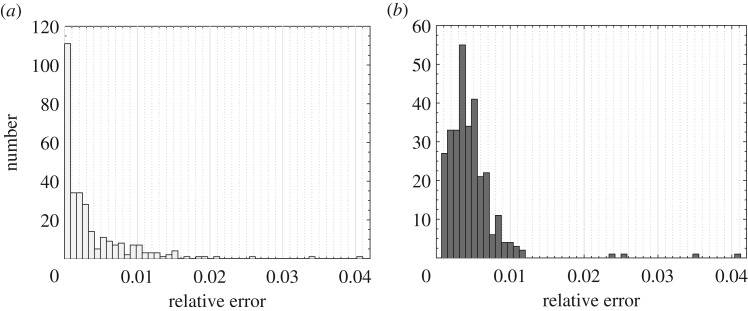
Histograms of *ϵ*_*r*_ corresponding to *m*=10, *r*=15 in figure 2, using: (*a*) kGPE-POD and (*b*) a global basis.

For *m*=10, kGPE-POD exhibits a minimum *ϵ*_*r*_ that is lower by more than an order of magnitude, while the maximum *ϵ*_*r*_ for both methods is roughly the same (0.04 for *r*≥15). At *r*=15 in figure 2*a*,*b*, the value of *ϵ*_*r*_ using kGPE-POD is lower than the minimum *ϵ*_*r*_ in the global basis method in 109 of the 300 test cases. For the global basis at *r*=15, there are 131 cases with an error below the median (3.9×10^−3^), while for kGPE-POD 217 cases have errors below this value. kGPE-POD clearly exhibits a broader range of *ϵ*_*r*_ values, with a higher median for *r*>25. Figure 3 shows histograms of *ϵ*_*r*_ for the two methods in the case of *r*=15, *m*=10. The broader range of *ϵ*_*r*_ is due to the much lower minimum and to the presence of a greater number of cases with *ϵ*_*r*_>0.012. The number of such cases (13) is, however, small. For *m*=100 snapshots, both methods improve, with the global basis method exhibiting the greater improvement (e.g. the maximum *ϵ*_*r*_ is decreased by around an order of magnitude, whereas for kGPE-POD the decrease is by a factor of 4 at *r*=15). The global basis method has a lower median *ϵ*_*r*_ for *r*≥20, but also again a considerably higher minimum (more than an order of magnitude at *r*=25). At *r*=30, for example, there are 77 cases in kGPE-POD with a lower *ϵ*_*r*_ than the minimum for the global basis.

**Figure 4.**
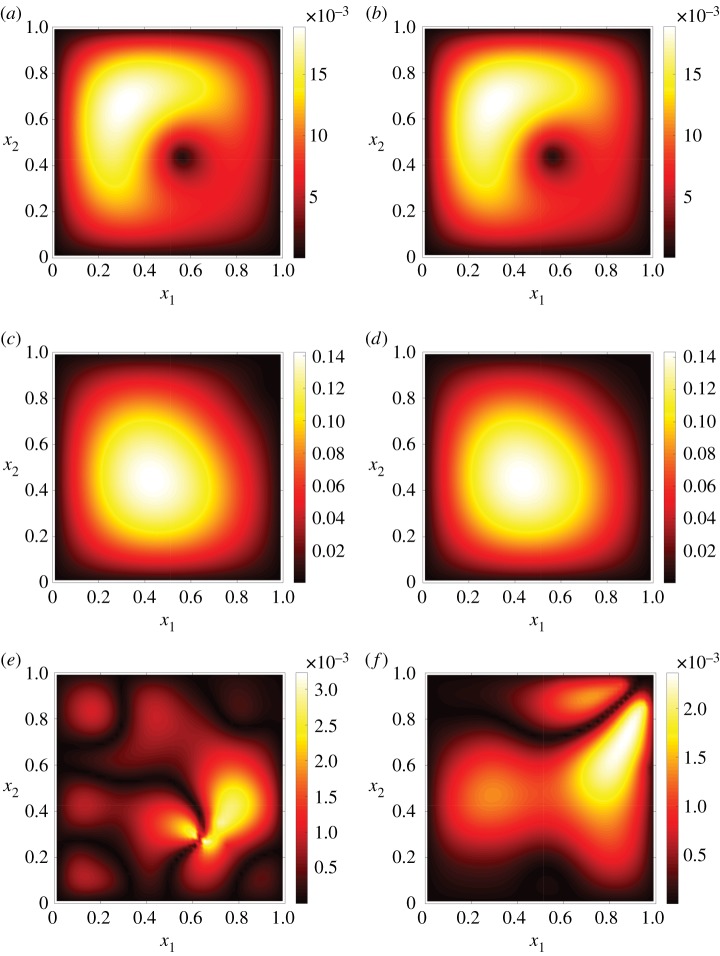
(*a*) The FOM and (*b*) the kGPE-POD prediction of the concentration field (mol m^−3^) for the contaminant transport model at ***ξ***=(0.7382,0.4179)^T^ and *t*=0.02*s* (*ϵ*_*r*_≈0.0021). (*c*) The FOM and (*d*) the kGPE-POD predictions at ***ξ***=(0.7539,0.7461)^T^ and *t*=0.2*s* (*ϵ*_*r*_≈0.0127). In all cases *n*=80, *m*=10 and *q*=6. (*e*) Absolute pointwise error for the case ***ξ***=(0.7382,0.4179)^T^ and (*f*) absolute pointwise error for ***ξ***=(0.7539,0.7461)^T^.

**Figure 5.**
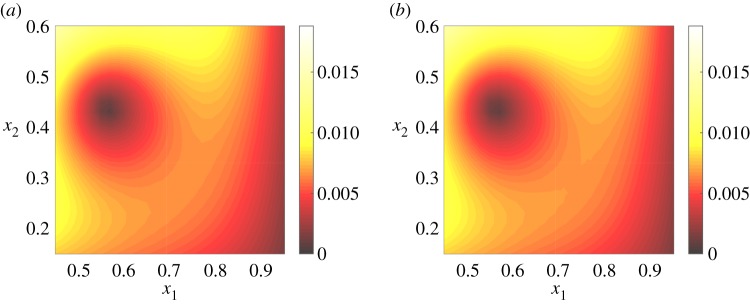
A close-up of (*a*) the kGPE-POD prediction and (*b*) the test corresponding to figure 4*a*,*b*.

To gain an indication of the actual quality of the predictions for different *ϵ*_*r*_, figure 4 compares the predicted kGPE-POD concentration fields in two test cases: (i) near the median (*ϵ*_*r*_≈0.0021) and (ii) near the upper whisker (*ϵ*_*r*_≈0.0127) at *r*=10 in figure 2*a*. The change in the profiles from one input to the other is well captured. Figure 4*e*,*f* shows the absolute pointwise errors for the two examples. It can be seen that there are localized regions of high error. For the first case (***ξ***=(0.7382,0.4179)^T^), a comparison of the region of highest error (lower right quadrant) with the test is shown in figure 5, which clearly highlights the fine-scale differences leading to the error. The trends and general profile (and in most of the domain the actual concentration values) are nevertheless well captured even with a small value of *r*.

**Figure 6.**
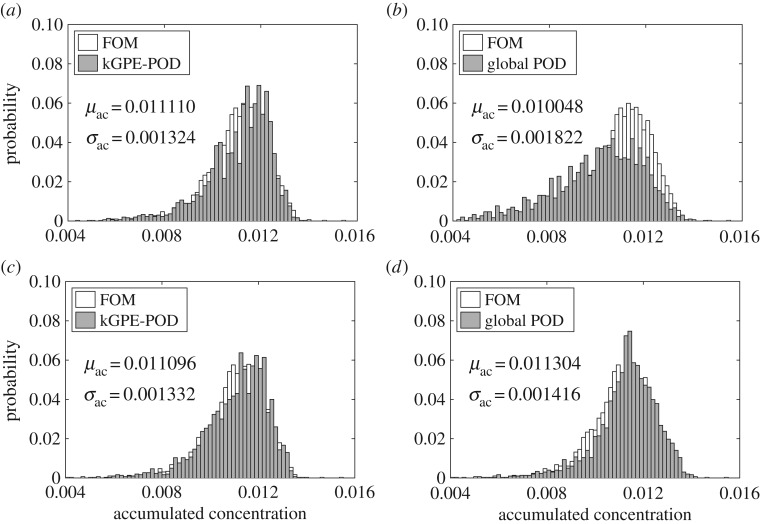
Estimated distribution of $\bar{u}({\text{x}}_{c};\xi )$ from *N*_*M*_=3000 Monte Carlo samples using *n*=80 and *m*=10: (*a*) kGPE-POD with *r*=10, (*b*) global basis with *r*=10, (*c*) kGPE-POD with *r*=50 and (*d*) global basis with *r*=50.

**Figure 7.**
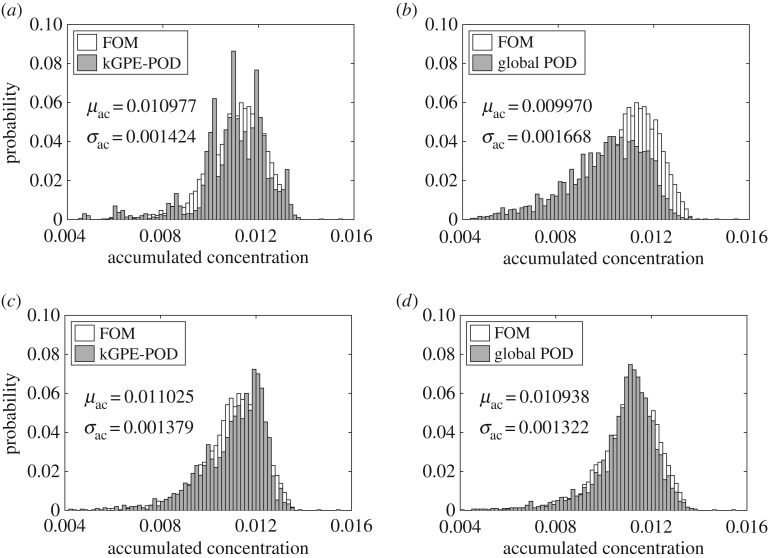
Estimated distribution of $\bar{u}({\text{x}}_{c};\xi )$ from *N*_*M*_=3000 Monte Carlo samples with *n*=80 and *m*=100: (*a*) kGPE-POD with *r*=10, (*b*) global basis with *r*=10, (*c*) kGPE-POD with *r*=30 and (*d*) global basis with *r*=30.

In order to assess the generalization accuracy more fully, we considered an uncertainty quantification problem for the accumulated contaminant concentration $\bar{u}(\text{x};\xi ):=\int_{0}^{T}u(\text{x},t;\xi )dt$ at the location ***x***_*c*_=(0.5,0.5)^T^, by considering ***ξ*** to be a random vector distributed according to $p(\xi )=N(\mu ,\sigma^{2}\text{I})$, where ***μ***=(0.5,0.5)^T^ and *σ*^2^=0.1. The distribution of $\bar{u}({\text{x}}_{c};\xi )$ was estimated using Monte Carlo sampling with *N*_*M*_ samples ***ξ***^*i*^ (this notation is to avoid confusion with the design points) drawn from *p*(***ξ***). We set *q*=6, *n*=80, *N*_*M*_=3000 and approximated $\bar{u}({\text{x}}_{c};\xi )$ with a trapezoidal rule. Figure 6 compares the histograms obtained from kGPE-POD, the global basis method and the FOM, using *m*=10 snapshots. The FOM took 55.18 h to complete and yielded *μ*_ac_=0.011087 and *σ*_ac_=0.001218, obtained from $\mu_{\mathrm{ac}}=(1/N_{M})\sum_{i=1}^{N_{M}}\bar{u}(\text{x};\xi^{i})$ and $\sigma_{\mathrm{ac}}^{2}={\left(N_{M}-1\right)}^{-1}\sum_{i=1}^{N_{M}}{\left(\bar{u}(\text{x};\xi^{i})-\mu_{\mathrm{ac}}\right)}^{2}$. For *r*=10, kGPE-POD exhibited reasonable accuracy with regards to *μ*_ac_ (within 0.2%) and *σ*_ac_ (within 8.7%), while the global basis method was inaccurate (50% error in *σ*_ac_). For *m*=10, *r*=50, both methods were accurate, with kGPE-POD still providing better estimates of *μ*_ac_ and *σ*_ac_. For *m*=100, the results are shown in figure 7. kGPE-POD was again more accurate for *r*=10, while for *r*=30 the two methods exhibited a similar level of accuracy.

### (b) Burgers equation

We consider a one-dimensional Burgers equation, with inputs ***ξ*** to be defined later:

4.4 $$\begin{array}{r} \partial_{t}u+\frac{1}{2}\partial_{x}(u^{2})-\frac{1}{Re}\partial_{xx}u=g(x),\;x\in D:=(0,1)\\ \mathrm{and}\;u(0,t)=u(1,t)=0,\;u(x,0)=u_{0}(x):=\sin(k\pi x)\;e^{-(c_{1}x+c_{2})}, \end{array}\}$$

where *u*(*x*,*t*;***ξ***) is the flow velocity, $c_{1},c_{2}\in R$, k∈N, *Re* is Reynold's number and *g*(*x*) is a source term. We seek a weak solution $u(x,t;\xi )\in V:=H_{0}^{1}(D)$ satisfying

4.5 $$(\partial_{t}u,v)+\frac{1}{2}(\partial_{x}(u^{2}),v)+\frac{1}{Re}a(u,v)=(g,v)\;\forall v\in V,$$

where *a*(*φ*_1_,*φ*_2_):=(*φ*_1_′,*φ*_2_′), $\varphi_{1},\varphi_{2}\in V$, defines a bilinear functional, in which a prime denotes an ordinary derivative w.r.t. *x*. The interval $\bar{D}=[0,1]$ is partitioned into *N*+1 equally sized subintervals [*x*_*i*_,*x*_*i*+1_], where *x*_*i*_=(*i*−1)/(*N*+1), *i*=1,…,*d*=*N*+2. A standard piecewise linear basis $\{\psi_{i}{\left(x)\right\}}_{i=1}^{d}$ defines the approximating space $V^{h}:=\text{span}(\psi_{1},\ldots ,\psi_{d})\subset V$.

The FE approximation $u(x,t;\xi )\approx u^{h}(x,t;\xi )=\sum_{j=1}^{d}u_{j}(t;\xi )\psi_{j}(x)$ leads to the weak formulation: find $u=u^{h}(x,t;\xi )\in V^{h}$ such that (4.5) holds $\forall v=v^{h}(x)\in V^{h}$. We also make use of the *group (product) approximation* [38]: $u{\left(x,t;\xi \right)}^{2}\approx \sum_{j=1}^{d}u_{j}{\left(t;\xi \right)}^{2}\psi_{j}(x)\in V^{h}$. Setting *u*=*u*^*h*^ and *v*^*h*^=*ψ*_*j*_ in (4.5), we obtain the semi-discrete problem

4.6 $$\sum_{i=1}^{d}{\dot{u\;}\;\;}_{i}(t;\xi )(\psi_{i},\psi_{j})+\frac{1}{2}\sum_{i=1}^{d}u_{i}{\left(t;\xi \right)}^{2}(\psi_{i}^{\prime },\psi_{j})+\frac{1}{Re}\sum_{i=1}^{d}u_{i}(t;\xi )(\psi_{i}^{\prime },\psi_{j}^{\prime })=(g,\psi_{j})$$

together with $\sum_{i=1}^{d}u_{i}(0;\xi )(\psi_{i},\psi_{j})=(u_{0},\psi_{j})$, ∀*j*=1,…,*d*. Defining the solution vector ***u***(*t*;***ξ***)=(*u*_1_(*t*;***ξ***),…,*u*_*d*_(*t*;***ξ***))^T^, equation (4.6) and the initial condition lead to

4.7 $$\text{M}\dot{\text{u}\;}\;\;(t;\xi )+\text{b}(\text{u}(t;\xi ))+\frac{1}{Re}\text{S}\text{u}(t;\xi )=\text{g},\;\text{M}\text{u}(0;\xi )={\text{u}}_{0},$$

where the (*i*,*j*)th elements of the mass and stiffness matrices **M** and **S** are given by (*ψ*_*i*_,*ψ*_*j*_) and (*ψ*_*i*_′,*ψ*_*j*_′), respectively, and the *j*th components of ***u***_0_ and **g** are (*u*_0_,*ψ*_*j*_) and (*g*,*ψ*_*j*_), respectively. The nonlinear vector function ***b***(***u***(*t*;***ξ***)) arises from the second term in (4.6). We used a Runge–Kutta method with a variable time step to solve the semi-discrete problems in this example.

The coefficients *u*_*i*,*j*_(***ξ***), *j*=1,…,*d*, of the snapshots ***u***_*i*_(***ξ***)=***u***(*t*_*i*_;***ξ***), *i*=1,…,*m*, for an arbitrary value of ***ξ*** are the nodal coefficients in the FE method solution, and thus correspond to functions ${\text{u}}_{i}(x,\xi ):=\sum_{j=1}^{d}u_{i,j}(\xi )\psi_{j}(x)\in V^{h}$. For the definition of the POD basis, we chose the $L^{2}(D)$ norm for optimality; that is, $H=L^{2}(D)$ as defined in appendix A. An FE approximation of the POD basis functions ${\left\{v_{j}^{h}(x;\xi )\right\}}_{j=1}^{d}$ is given by $v_{j}^{h}(x;\xi )=\sum_{i=1}^{d}v_{j,i}(\xi )\psi_{i}(x)\in V^{h}$, *j*=1,…,*d*, in which the nodal coefficient *v*_*j*,*i*_(***ξ***) is the *i*th component of the POD basis vector ***v***_*j*_(***ξ***), given by ${\text{v}}_{j}(\xi )={\text{M}}^{-1/2}{\bar{\text{v}}}_{j}(\xi )$, where ${\bar{\text{v}}}_{j}(\xi )$ is an eigenvector of **M**^1/2^**C**(***ξ***)**M**^1/2^. Note that $L^{2}(D)$ orthogonality of the basis ${\left\{v_{j}^{h}(x;\xi )\right\}}_{j=1}^{d}$ is equivalent to orthogonality of the ***v***_*j*_(***ξ***) w.r.t. 〈***v***_*j*_(***ξ***),***v***_*i*_(***ξ***)〉_**M**_:=***v***_*j*_(***ξ***)^T^**M*v***_*i*_(***ξ***). The solution vector is then expanded as in equations (2.3): $\text{u}(t;\xi )\approx \text{u}(t;\xi )=\sum_{j=1}^{r}a_{j}(\text{t};\xi ){\text{v}}_{j}(\xi )={\text{V}}_{r}(\xi )\text{a}(t;\xi )$, leading to the ROM

4.8 $$\begin{array}{r} \dot{\text{a}\;}\;\;(t;\xi )+{\text{V}}_{r}{\left(\xi \right)}^{T}\text{b}\left({\text{V}}_{r}(\xi )\text{a}(t;\xi )\right)+\frac{1}{Re}{\text{V}}_{r}{\left(\xi \right)}^{T}\text{S}{\text{V}}_{r}(\xi )\text{a}(t;\xi )={\text{V}}_{r}{\left(\xi \right)}^{T}\text{g}\\ \text{and}\;\text{a}(0;\xi )={\text{a}}_{0}(\xi ):={\text{V}}_{r}{\left(\xi \right)}^{T}{\text{u}}_{0}. \end{array}\}$$

Another choice for optimality is $H=H_{1}^{0}(D)$ with *a*(⋅,⋅) as the inner product and associated semi-norm |*φ*|_1_=*a*(*φ*,*φ*)^1/2^. The POD eigenvalue problem $\int_{0}^{T}a(u,v)u\;dt=\lambda v$ (see appendix A) leads to the eigenvalue problem **C**(***ξ***)^T^**S*v***_*j*_(***ξ***)=*λ****v***_*j*_(***ξ***). The POD basis vectors are then given by ${\text{v}}_{j}(\xi )={\text{S}}^{-1/2}{\bar{\text{v}}}_{j}(\xi )$, where ${\bar{\text{v}}}_{j}(\xi )$ is an eigenvector of **S**^1/2^**C**(***ξ***)**S**^1/2^, and are mutually orthogonal w.r.t. 〈⋅,⋅〉_**S**_. In the present example, this approach gave almost identical results.

*Case* 1. In the first example, we set *g*(*x*)≡0 and *k*=1. The inputs were defined as $\xi ={\left(c_{1},c_{2},Re\right)}^{T}\in X=[2,5]\times [0.1,1]\times [10,1000]$. A total of 500 inputs $\xi_{j}\in X$ were selected using a Sobol sequence and numerical experiments were performed by solving the FOM (4.7) with *d*=64 nodes, for each *j*=1,…,500, to obtain the solution vectors ***u***(*t*_*i*_;***ξ***_*j*_) and nonlinearity ***b***(***u***(*t*_*i*_;***ξ***_*j*_)) at times *t*_*i*_=0.25*i*, *i*=1,…,40 (*m*=40). This yielded the data points (vectorized snapshots) ***y***_*j*_=***η***(***ξ***_*j*_) and ${\text{y}}_{j}^{f}=\eta^{f}(\xi_{j})$, *j*=1,…,500, according to equation (3.3). Of the 500 data points, *n*_*t*_=300 were reserved for testing, and training points were selected from the remaining 200 points. The details of kPCA and GPE were as described in the previous example.

**Figure 8.**
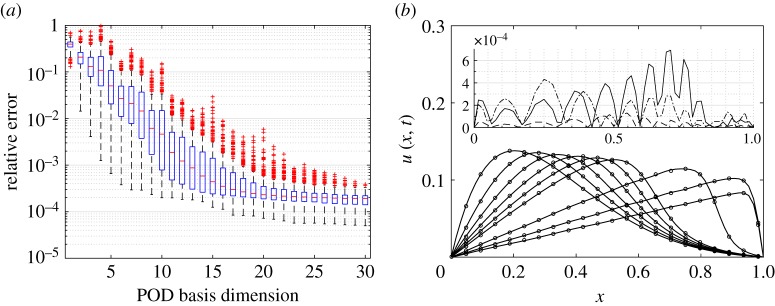
(*a*) Tukey box plots of *ϵ*_*r*_ with increasing *r* using kGPE-POD-DEIM for Burgers model case 1 (*n*=180, *n*_*t*_=300 and *m*=15). (*b*) Velocity profiles at *t*=0,0.5,1,1.5,2,2.5,5,7.5,10 s simulated with the FOM (filled circles, every third node) and kGPE-POD-DEIM (solid lines) for a case with *ϵ*_*r*_≈0.041 at *r*=10. The inset in panel *b* shows the absolute pointwise error at *t*=2.5 s (dashed), 5 s (solid) and 10 s (dashed-dotted). (Online version in colour.)

Analysis of the normalized errors *ϵ* for the *n*_*t*_ test cases with *n*=160 training points showed convergence after *q*=8 dimensions. Isomap gave similar results while Diffusion maps was inferior. We used *q*=9 (kPCA) in the results presented below. Figure 8*a* shows the results of kGPE-POD-DEIM for an increasing *r* (with *s*=*r*). The relative errors converge after *r*=30, i.e. further decreases are negligible. Figure 8*b* compares the predicted velocity profiles at *t*=0,0.5,1,1.5,2,2.5,5,7.5,10 s from kGPE-POD-DEIM and the FOM for a point (*ϵ*_*r*_≈0.041) above the upper whisker at *r*=10 in figure 8*a*. The two sets of profiles are very close. The inset in figure 8*b* shows the absolute pointwise error at *t*=2.5, 5 and 10 s. Inspection of the full set of profiles showed that the error grew with time until the front developed, after which the error decayed. The highest absolute error was around 8.62×10^−4^ at *x*=0.703, *t*=5.65 s, for which *u*(*x*,*t*)≈0.103 m s^−1^. Thus, the maximum error was around 0.84%.

**Figure 9.**
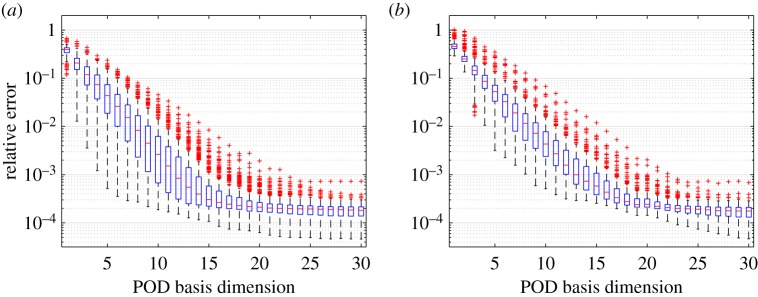
Tukey box plots of *ϵ*_*r*_ with increasing *r* for Burgers model case 1 (*n*_*t*_=300, *m*=40 and *n*=180): (*a*) kGPE-POD and (*b*) a global basis. (Online version in colour.)

With no approximation of the nonlinearity, a comparison between kGPE-POD and the global basis method exhibited trends similar to those seen in the previous example. For *m*<30 and *n*≤200, kGPE-POD required fewer POD vectors to achieve a given level of accuracy; the lower bound for *ϵ*_*r*_ at *r*=10 was one order of magnitude smaller for kGPE-POD. Both methods improved with increasing *m*, with the global basis method showing a greater improvement, especially in the lower bound for *ϵ*_*r*_. For *m*=30 and *n*=180 the results are illustrated in figure 9, which shows that around *r*=28 both methods exhibit similar levels of accuracy in terms of the maximum, minimum and median *ϵ*_*r*_.

*Case* 2. In a second case, we set *g*(*x*)=0.02*e*^*x*^, *k*=3 and *c*_2_=0.2, with inputs ξ=(c1,Re)T∈X=[2,5]×[10,1000]. As before we selected 500 inputs using a Sobol sequence and ran the FOM to generate data points, with *n*_*t*_=300 reserved for testing. In this case, we use *d*=128 nodes and after inspection of the normalized errors *ϵ* we set *q*=9. In contrast to the previous case, a large *m* (*m*>120) was required for accurate results.

**Figure 10.**
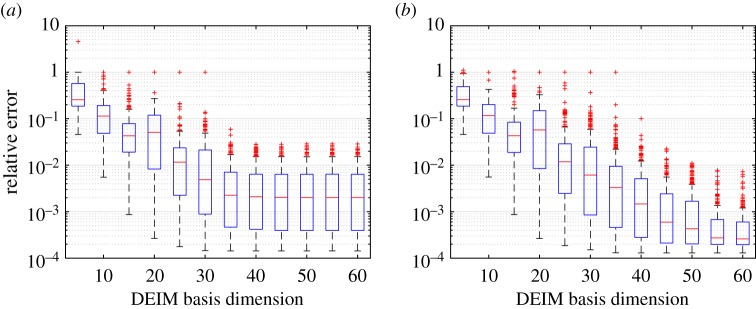
Tukey box plots of *ϵ*_*r*_ with increasing *s* for Burgers model case 2 (*n*_*t*_=300, *n*=180 and *m*=200) using kGPE-POD-DEIM with: (*a*) *r*=30 and (*b*) *r*=50. (Online version in colour.)

Figure 10 shows the trends in the kGPE-POD-DEIM relative error *ϵ*_*r*_ on the *n*_*t*_=300 test points with increasing *s* for two values of *r*, using *n*=180 and *m*=200. For a fixed *r*, the errors decrease with an increasing *s*. For a fixed *s*, the errors were seen to decrease as *r* was increased up to a certain value. For higher values of *r* the solutions became less stable, with a corresponding increase in the error. This was more pronounced for small values of *s*. The optimal distribution of errors (in terms of the median, quartiles and extrema) was achieved for values of *s* between 5 and 10 higher than the value of *r*. Similar results for Burgers equation can be found in [39,40].

**Figure 11.**
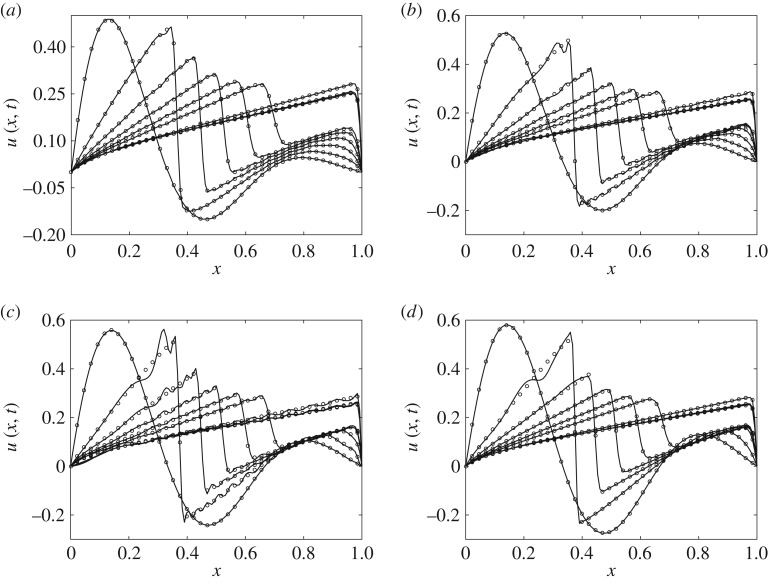
Velocity profiles predicted by the FOM (filled circles, every third node) and kGPE-POD-DEIM (solid lines) at *t*=0,0.5,1,1.5,2,2.5,5,7.5,10 s for Burgers model case 2. (*a*) A point near the median (*ϵ*_*r*_≈0.0022) at *r*=30, *s*=40 in figure 10*a*; (*b*) a point near the upper whisker (*ϵ*_*r*_≈0.0154) at *r*=30, *s*=40; (*c*) point with the highest error (*ϵ*_*r*_≈0.0282) at *r*=30, *s*=40; (*d*) point with the highest error (*ϵ*_*r*_≈0.0072) at *r*=50, *s*=55 in figure 10*b*.

For *r*=30 and *s*=40, figure 11*a*,*b* compares the FOM and kGPE-POD-DEIM profiles at *t*=0,0.5,1,1.5,2,2.5,5,7.5,10 s. The first of these corresponds to a point near the median of the relevant box plot in figure 10*a*, while the second corresponds to a point near the upper whisker. Figure 11*c* shows the point with the highest error using the same values of *r* and *s*. In this case, instability develops as the front forms but eventually settles. Using *r*=50 and *s*=55, the case with the highest error is shown in figure 11*d*. In figure 11*d*, we see that the solutions at early times are more stable. The observed instability is a common feature of POD models [27,41,42]. Stabilization schemes, e.g. alternative inner products, post-processing steps and modification of the underlying model [42–44], can be incorporated within the framework we have developed in order to eliminate or minimize such problems.

## 5. Conclusion

In this paper, we introduced a new POD-ROM method for dynamic, parameter-dependent linear and nonlinear PDEs. The method uses a Bayesian nonlinear regression to infer the basis for new parameter values and is thus potentially applicable to a broader window of parameter space than existing methods. Compared with a global POD method, our method requires extra computational effort on each run to diagonalize the snapshot matrix. The actual influence of this is small (as the uncertainty quantification in the first example demonstrates) since most of the computational time is spent on solving the ROM. In the examples considered (and others not presented) the global basis method requires a high value of *r* to reach the same level of performance (in terms of the minimum and maximum relative errors) as our method, particularly for low values of *m*. At these high values of *r*, much of the benefits of the global basis method as a surrogate model would be eliminated.

Since the method introduced here is a general framework, a number of modifications could easily be made, e.g. changing the manifold learning or machine learning method, and incorporating stabilization schemes, according to different types of problems. The manifold-learning-based GP emulator could be treated as a general data-driven technique to interpolate properties other than the snapshots, as has been accomplished with the method of Amsallem & Farhat [14]. For instance, we could employ it to directly learn the POD basis **V**_*r*_(***ξ***) in equation (2.3) or the system matrix **A**_*r*_(***ξ***) in equation (2.4), both of which would further reduce the computational time.

## Appendix A. Variants of POD

We regard *u*(***x***,*t*;***ξ***) as a realization of a zero-mean random field indexed by (***x***,*t*) [3,4,29], with an underlying probability space (Ω,A,P), where *Ω* is the sample space, A is the event space and P is a probability measure. It is assumed that *u*(***x***,*t*;***ξ***) is continuous in quadratic mean (q.m.) w.r.t. ***x*** (assumption (i)) and stationary w.r.t. *t* (assumption (ii)). The spatial autocovariance function then takes the form $E[u(\text{x},t;\xi )u({\text{x}}^{\prime },t;\xi )]=C(\text{x},{\text{x}}^{\prime };\xi )$, $\text{x},{\text{x}}^{\prime }\in D$. For a fixed *t*∈[0,*T*], *u*(***x***,*t*;***ξ***) defines a *one-parameter* random field indexed by x∈D [29]. Sample paths (fixed *ω*∈*Ω*) are deterministic functions u(⋅,t;ξ):D→R. By assumption (i), $u(\cdot ,t;\xi )\in L^{2}(D)$ for each *t*∈[0,*T*], so that $u(\text{x},t;\xi )\in L^{2}(0,T;L^{2}(D))$. KL theory [30] for a fixed *t* shows that *u*(***x***,*t*;***ξ***) is the q.m. limit of the sequence of partial sums ${\text{S}}_{M}=\sum_{i=1}^{M}a_{i}(t;\xi )v_{i}(\text{x};\xi )$, with randomness entering only through *t*. The *v*_*i*_(***x***;***ξ***) form an $L^{2}(D)$ orthonormal basis and are given by the eigenfunctions of an operator C,

A 1 $$Cv_{i}(\text{x};\xi ):=\int_{D}C(\text{x},{\text{x}}^{\prime };\xi )v_{i}({\text{x}}^{\prime };\xi )\;d{\text{x}}^{\prime }=\lambda_{i}^{\prime }v_{i}(\text{x};\xi )\;i\in N$$

with corresponding real, positive eigenvalues *λ*_*i*_′(***ξ***)>*λ*_*i*+1_′(***ξ***) ∀i∈N. The coefficients satisfy $E[a_{i}(t;\xi )]=0$ and $E[a_{i}(t;\xi )a_{j}(t;\xi )]=\lambda_{i}^{\prime }(\xi )\delta_{ij}$ and, since *t* is arbitrary, they can be interpreted as uncorrelated random processes. The expectation operator $E[X]=\int_{\Omega }X(\omega )P(d\omega )$ is approximated by a time average (obtained from the snapshots), assuming ergodicity.

The ‘optimality’ of the basis ${\left\{v_{i}(\text{x};\xi )\right\}}_{i\in N}$ can be interpreted in two equivalent ways. For an arbitrary orthonormal basis ${\left\{\varphi_{i}\right\}}_{i=1}^{\infty }$ of $L^{2}(D)$, $\sum_{i=1}^{r}E[{\left(u,v_{i}\right)}^{2}]=\sum_{i=1}^{r}\lambda_{i}^{\prime }>\sum_{i=1}^{r}E[{\left(u,\varphi_{i}\right)}^{2}]$, ∀r∈N, i.e. a generalized ‘variance’ maximization. Equivalently, we minimize the ‘error’ $E[∥u-\sum_{i=1}^{r}a_{i}\varphi_{i}{∥}^{2}]=∥u-\sum_{i=1}^{r}a_{i}\varphi_{i}{∥}_{L^{2}(0,T;L^{2}(D))}^{2}$ over orthonormal bases ${\left\{\varphi_{i}\right\}}_{i=1}^{\infty }$. These results carry over to the finite-dimensional setting described below, in which case orthonormality is defined w.r.t. an inner product in $R^{d}$. More generally, we seek $\min_{\left\{\varphi_{i}\right\}}∥u-\sum_{i=1}^{r}a_{i}\varphi_{i}{∥}_{L^{2}(0,T;H)}^{2}$ for any separable Hilbert space H. In this case, the POD basis is defined by the H-orthonormal eigenfunctions v(x)∈H of the operator $Rv:=E[u{\left(u,v\right)}_{H}]=\int_{0}^{T}u{\left(u,v\right)}_{H}\;dt$. For $H=L^{2}(D)$, R=C using the commutativity of the time and spatial averaging operations.

Defining quadrature points ${\left\{t_{i}\right\}}_{i=1}^{m}$ and (equally spaced) ${\left\{{\text{x}}_{i}\right\}}_{i=1}^{d}$, problem (A 1) can be approximated numerically using a midpoint rule: **C**(***ξ***)***v***_*i*_(***ξ***)=*λ*_*i*_(***ξ***)***v***_*i*_(***ξ***) for eigenvectors ${\text{v}}_{i}(\xi )\in R^{d}$ and positive (decreasing) eigenvalues *λ*_*i*_(***ξ***), *i*=1,…,*d*. This is a PCA with a spatial variance–covariance matrix $\text{C}(\xi )=\text{U}(\xi )\text{U}{\left(\xi \right)}^{T}\approx E[\text{u}(t;\xi )\text{u}{\left(t;\xi \right)}^{T}]$, in which **U**(***ξ***):=[***u***_1_(***ξ***)…***u***_*m*_(***ξ***)]. The *j*th component *v*_*i*,*j*_(***ξ***) of ***v***_*i*_(***ξ***) can be identified with *v*_*i*_(***x***_*j*_;***ξ***) and the (*i*,*j*)th entry of **C**(***ξ***) approximates *C*(***x***_*i*_,***x***_*j*_;***ξ***) as the time average $\left(1/m)\sum_{k}u({\text{x}}_{i},t_{k};\xi )u({\text{x}}_{j},t_{k};\xi \right)$. Other interpolation procedures for (A 1) can also be used. For instance, in the FE formulation (§4b) we can approximate *u*(***x***,*t*;***ξ***) and *v*_*i*_(***x***;***ξ***) using a standard piecewise linear basis ${\left\{\psi_{i}(\text{x})\right\}}_{i=1}^{d}\subset L^{2}(D)$, which leads to **C**(***ξ***)**M*****v***_*i*_(***ξ***)=*λ*_*i*_(***ξ***)***v***_*i*_(***ξ***), where **M** is a mass matrix with entries *M*_*ij*_=(*ψ*_*i*_(***x***),*ψ*_*j*_(***x***)). Defining $\bar{\text{v}}(\xi )={\text{M}}^{1/2}\text{v}(\xi )$ we obtain ${\text{M}}^{1/2}\text{C}(\xi ){\text{M}}^{1/2}\bar{\text{v}}(\xi )=\lambda (\xi )\bar{\text{v}}(\xi )$. The eigenvalue/eigenvector pairs ${\left\{({\bar{\text{v}}}_{i}(\xi ),\lambda_{i}(\xi ))\right\}}_{i=1}^{d}$ of **M**^1/2^**C**(***ξ***)**M**^1/2^ yield the POD basis vectors ${\text{v}}_{i}(\xi )={\text{M}}^{-1/2}{\bar{\text{v}}}_{i}(\xi )$ in the desired order.

The *method of snapshots* is used when *m*≪*d*. The temporal autocovariance function $K(t,t^{\prime };\xi )=\int_{D}u(\text{x},t;\xi )u(\text{x},t^{\prime };\xi )\;d\text{x}$ defines an operator $K{\text{a}}_{i}(t;\xi ):=\int_{0}^{T}K(t,t^{\prime };\xi ){\text{a}}_{i}(t^{\prime };\xi )\;dt^{\prime }$. The eigenfunctions *a*_*i*_(*t*;***ξ***) of K are equal to the POD coefficients, and the eigenvalues are identical to those of C. Using $E[a_{i}(t;\xi )a_{j}(t;\xi )]=\lambda_{i}^{\prime }(\xi )\delta_{ij}$, the POD modes are given by $v_{i}(\text{x};\xi )=(1/\lambda_{i}^{\prime }(\xi ))\int_{0}^{T}u(\text{x},t;\xi )a_{i}(t;\xi )\;dt$. The discrete form (in space and time) of the eigenvalue problem is **U**(***ξ***)^T^**U**(***ξ***)***a***_*i*_(***ξ***)=*λ*_*i*_***a***_*i*_(***ξ***), where **K**(***ξ***):=**U**(***ξ***)^T^**U**(***ξ***) is a kernel matrix with entries *K*_*ij*_=***u***_*i*_(***ξ***)^T^***u***_*j*_(***ξ***), i.e. the space-discrete form of *K*(*t*_*i*_,*t*_*j*_;***ξ***). The eigendecomposition is **K**(***ξ***)=**A**(***ξ***)***Λ***(***ξ***)**A**(***ξ***)^T^, where ***Λ***(***ξ***)=diag(*λ*_1_(***ξ***),…,*λ*_*m*_(***ξ***)) and the columns of **A**(***ξ***) are given by the ***a***_*i*_(***ξ***). The *j*th component *a*_*i*,*j*_(***ξ***) of ***a***_*i*_(***ξ***) approximates *a*_*i*_(*t*_*j*_;***ξ***), yielding the discrete-time approximation $v_{i}(\text{x};\xi )=(1/\lambda_{i}(\xi ))\sum_{j=1}^{m}u(\text{x},t_{j};\xi )a_{i,j}(\xi )$, i.e. a linear combination of the snapshots. In the fully discrete case, using the normalization ${\text{a}}_{i}(\xi )\mapsto {\text{a}}_{i}^{\prime }(\xi )/\sqrt{\lambda_{i}(\xi )}$, we obtain ${\text{v}}_{i}(\xi )=\text{U}(\xi ){\text{a}}_{i}^{\prime }(\xi )/\sqrt{\lambda_{i}(\xi )}$. These relationships are also evident from the SVD of **U**(***ξ***), that is, **U**(***ξ***)=**A**′(***ξ***)***Λ***(***ξ***)^1/2^**V**(***ξ***)^T^, where the columns of **V**(***ξ***) are given by the ***v***_*i*_(***ξ***) and the columns of **A**′(***ξ***) are given by the ***a***_*i*_′(***ξ***). In this context, the columns of **A**′(***ξ***) and **V**(***ξ***), given, respectively, by the eigenvectors of **K**(***ξ***) and **C**(***ξ***), are referred to as the left and right singular vectors. It is straightforward to show that ***v***_*i*_(***ξ***)=*k***U**(***ξ***)***a***_*i*_′(***ξ***) for k∈R. Thus, we recover the earlier relationship by taking $k=1/\sqrt{\lambda_{i}(\xi )}$ to normalize the ***v***_*i*_(***ξ***).

## Appendix B. Kernel principal component analysis and an inverse mapping

**(a) Kernel principal component analysis on the training data**

Given training data ${\text{y}}_{i}=\eta (\xi_{i})\in O$, *i*=1,…,*n*, kPCA [35] defines a map ϕ:O→F, where F is a feature space. The mapped points are centred by $\tilde{\phi }({\text{y}}_{i})=\phi ({\text{y}}_{i})-\bar{\phi }$, where $\bar{\phi }=n^{-1}\sum_{j=1}^{n}\phi ({\text{y}}_{j})$. The ‘tilde’ symbol is used throughout to denote the corresponding quantity centred in feature space. kPCA then applies linear PCA to the sample covariance matrix ${\text{C}}_{F}=n^{-1}\sum_{i=1}^{n}\tilde{\phi }({\text{y}}_{i}){\tilde{\phi }({\text{y}}_{i})}^{T}$. The map ***ϕ***(⋅) is specified implicitly via a kernel function *k*(***y***_*i*_,***y***_*j*_)=***ϕ***(***y***_*i*_)^T^***ϕ***(***y***_*j*_), e.g. the Gaussian kernel $k({\text{y}}_{i},{\text{y}}_{j})=\exp\left(-∥{\text{y}}_{i}-{\text{y}}_{j}{∥}^{2}/s^{2}\right)$, where *s* is a scale factor. Defining a kernel matrix $\text{K}={\left[K_{ij}=k({\text{y}}_{i},{\text{y}}_{j})\right]}_{i,j=1}^{n}$, a centred kernel matrix is given by $\tilde{\text{K}}=\text{H}\text{K}\text{H}$, where **H**=**I**−*n*^−1^**1****1**^T^ and $\text{1}=n^{-1}{\left(1,\ldots ,1\right)}^{T}\in R^{n}$.

We assume that dimF>n without loss of generality. The orthonormal eigenvectors ***w*** of ${\text{C}}_{F}$ are linear combinations of $\tilde{\phi }({\text{y}}_{i})$ and the eigenvalue problem for ${\text{C}}_{F}$ is equivalent to the eigenvalue problem for $n^{-1}\tilde{\text{K}}$ [35], which possesses orthonormal eigenvectors ***α***_*i*_=(*α*_1*i*_,…,*α*_*ni*_)^T^, *i*=1,…,*n*. The common eigenvalues *β*_*i*_ of $n^{-1}\tilde{\text{K}}$ and ${\text{C}}_{F}$ are arranged in decreasing order. The rescaling $\alpha_{i}\mapsto {\hat{\alpha }}_{i}:=\alpha_{i}/\sqrt{\beta_{i}}$ yields rescaled eigenvectors ${\hat{\text{w}}}_{i}=\sum_{j=1}^{n}{\hat{\alpha }}_{ji}\tilde{\phi }({\text{y}}_{j})$, where ${\hat{\alpha }}_{ji}=\alpha_{ji}/\sqrt{\beta_{i}}$. We can now write $\tilde{\phi }({\text{y}}_{j})=\sum_{i=1}^{n}z_{i}({\text{y}}_{j}){\hat{\text{w}}}_{i}$, where

B 1 $$z_{i}({\text{y}}_{j})={\hat{\text{w}}}_{i}^{T}\tilde{\phi }({\text{y}}_{j})=\sum_{l=1}^{n}{\hat{\alpha }}_{li}{\tilde{K}}_{lj}={\hat{\alpha }}_{i}^{T}{\tilde{\text{k}}}_{j}={\hat{\alpha }}_{i}^{T}\text{H}({\text{k}}_{j}-\text{K}\text{1}),\;i=1,\ldots ,n,$$

in which ***k***_*j*_=(*K*_1*j*_,…,*K*_*nj*_)^T^ and ${\tilde{\text{k}}}_{j}={\left({\tilde{K}}_{1j},\ldots ,{\tilde{K}}_{nj}\right)}^{T}$. From equation (B 1), we can define ${\text{z}}_{q}({\text{y}}_{j}):={\left(z_{1}({\text{y}}_{j}),\ldots ,z_{q}({\text{y}}_{j})\right)}^{T}={\left[{\hat{\alpha }}_{1}\ldots {\hat{\alpha }}_{q}\right]}^{T}\text{H}({\text{k}}_{j}-\text{K}\text{1})$, where *q*<*n* is the approximate dimension of the manifold on which the points reside.

The variance in the data along ${\hat{\text{w}}}_{i}$ is equal to *β*_*i*_, which decreases as *i* increases, and the coefficients *z*_*i*_(⋅) are mutually uncorrelated [45]. A point ***y***_*j*_ is approximated by the projection of its image $\tilde{\phi }({\text{y}}_{j})$ onto the low-dimensional subspace $F_{q}=\text{span}({\hat{\text{w}}}_{1},\ldots ,{\hat{\text{w}}}_{q})\subset F$. The projection is defined by ${\tilde{\phi }}_{q}(\cdot ):=\sum_{i=1}^{q}z_{i}(\cdot ){\hat{\text{w}}}_{i}$. The 2-norm error in this approximation is equal to $\sum_{i=q+1}^{n}\beta_{i}^{2}$ [45]. We use ${\left\{{\hat{\text{w}}}_{i}\right\}}_{i=1}^{n}$ as an approximate basis for the image $\tilde{\phi }[O]\subset F$ of all points in O. For an arbitrary y∈O, a reduced-dimensional approximation is given by ${\tilde{\phi }}_{q}(\text{y})=\sum_{i=1}^{q}z_{i}(\text{y}){\hat{\text{w}}}_{i}$. We can now define the composite maps ${\text{ℤ}}_{i}(\cdot ):=z_{i}(\eta (\cdot )):X\to R^{r}$, *i*=1,…,*n*.

**(b) Inverse map**

To define an approximate inverse map $\phi_{q}^{-1}:F_{q}\to O$, we use a weighted average of *N*_*n*_≤*n* ‘neighbouring’ points of ***y*** taken from the dataset: $\text{y}=\sum_{j\in J}\rho ({\text{y}}_{j}){\text{y}}_{j}$, with weights *ρ*(***y***_*j*_), where J⊆{1,2,…,n} defines the neighbouring points. We can define the weights in terms of the distances *d*_*j*,*_, *j*=1,…,*n*, between ***y*** and ***y***_*j*_, and use an isotropic kernel density *χ*(***y***,***y***′)=*χ*(∥***y***−***y***′∥) to weight the samples [34]: $\rho ({\text{y}}_{j})=\chi (d_{j,*})/\sum_{i\in J}\chi (d_{i,*})$. In this paper, we use $\chi (\text{y},{\text{y}}^{\prime })=\exp(-∥\text{y}-{\text{y}}^{\prime }{∥}^{2})$ [34]. Since $\tilde{\Phi }=\Phi \text{H}$, where *Φ*=[***ϕ***(***y***_1_),…,***ϕ***(***y***_*n*_)], we have ${\hat{\text{w}}}_{i}=\sum_{j=1}^{n}{\hat{\alpha }}_{ji}\tilde{\phi }({\text{y}}_{j})=\tilde{\Phi }{\hat{\alpha }}_{i}=\Phi \text{H}{\hat{\alpha }}_{i}$, which yields

B 2 $$\phi_{q}(\text{y})=\sum_{i=1}^{q}z_{i}{\hat{\text{w}}}_{i}+\bar{\phi }\sum_{i=1}^{q}z_{i}\Phi \text{H}{\hat{\alpha }}_{i}+\Phi \text{1}\Phi (\text{H}[{\hat{\alpha }}_{1}\ldots ,{\hat{\alpha }}_{q}]{\text{z}}_{q}+\text{1})=\Phi \tau .$$

The distance ${\tilde{d}}_{j,*}$ between ***ϕ***(***y***_*j*_) and ***ϕ***(***y***) in F is given by

B 3 $${\tilde{d}}_{j,*}^{2}=\phi {\left(\text{y}\right)}^{T}\phi (\text{y})+\phi {\left({\text{y}}_{j}\right)}^{T}\phi ({\text{y}}_{j})-2\phi {\left(\text{y}\right)}^{T}\phi ({\text{y}}_{j}).$$

Taking ***ϕ***(***y***)≈***ϕ***_*q*_(***y***) and substituting equation (B 2) into equation (B 3) yields

B 4 $${\tilde{d}}_{j,*}^{2}\approx \tau^{T}\text{K}\tau +k({\text{y}}_{j},{\text{y}}_{j})-2\tau^{T}{\text{k}}_{j}.$$

Note that *Φ*^T^*Φ*=**K** and ***k***_*j*_=*Φ*^T^***ϕ***(***y***_*j*_). For an isotropic kernel normalized such that *k*(***y***′,***y***′)=1, equation (B 3) gives ${\tilde{d}}_{j,*}^{2}=2-2k({\text{y}}_{j},\text{y})$, which, equating to the right-hand side of equation (B 4), yields *k*(***y***_*j*_,***y***). For the Gaussian kernel, therefore, we obtain $d_{j,*}^{2}=-s^{2}\ln k({\text{y}}_{j},\text{y})$.

## Appendix C. Gaussian process emulation

We place independent GP priors over the coefficients ${\text{ℤ}}_{i}(\xi )$ and emulate each independently, using training data obtained from equation (B 1), i.e. ${\left\{{\text{ℤ}}_{i}(\xi_{j})={\hat{\alpha }}_{i}^{T}\text{H}({\text{k}}_{j}-\text{K}\text{1})\right\}}_{j=1}^{n}$, *i*=1,…,*q*. For any value of *i*, let $\eta (\cdot ):={\text{ℤ}}_{i}(\cdot )$. Then $\eta (\xi_{j})={\text{ℤ}}_{i}(\xi_{j})$, *j*=1,…,*n*, and we define ***t***:=(*η*(***ξ***_1_),…,*η*(***ξ***_*n*_))^T^. A univariate GP prior indexed by ξ∈X is placed over *η*, namely, $\eta (\xi )|\theta ∼GP(m(\xi ),\text{ℂ}(\xi ,\xi^{\prime };\theta ))$, where GP(m(⋅),ℂ(⋅,⋅;θ)) represents a GP with mean and covariance functions *m*(⋅) and ℂ(⋅,⋅;θ), respectively. We take *m*(***ξ***)≡0 by centring the data ${\left\{\eta (\xi_{i})\right\}}_{j=1}^{n}$. The quantity ***θ*** is a vector of hyperparameters that appear in the covariance function and typically have to be inferred from the data. We use a square exponential covariance function

C 1 $$\text{ℂ}(\xi ,\xi^{\prime };\theta )=\theta_{0}\exp(-{\left(\xi -\xi^{\prime }\right)}^{T}\;\text{diag}(\theta_{1},\ldots ,\theta_{l})(\xi -\xi^{\prime }))+\sigma_{n}^{2}\delta (\xi ,\xi^{\prime }),$$

where the last term is a GP noise and $\theta ={\left(\theta_{0},\ldots ,\theta_{l},\sigma_{n}^{2}\right)}^{T}$ is the vector of hyperparameters, in which *θ*_1_,…,*θ*_*l*_ are the inverse square correlation lengths. The conditional predictive distribution is obtained from *p*(*η*(***ξ***),***t***|***θ***) in the form $\eta (\cdot )|\text{t},\theta ∼GP(m^{\prime }(\cdot ;\theta ),\nu^{\prime }(\cdot ,\cdot ;\theta ))$, with [46],

C 2 $$m^{\prime }(\xi ;\theta )=c{\left(\xi \right)}^{T}{\text{C}}^{-1}\text{t},\;\nu^{\prime }(\xi ,\xi^{\prime };\theta )=\text{ℂ}(\xi ,\xi^{\prime };\theta )-c{\left(\xi \right)}^{T}{\text{C}}^{-1}c(\xi^{\prime }),$$

where $\text{C}={\left[\text{ℂ}(\xi_{i},\xi_{j};\theta )\right]}_{i,j=1}^{n}$ and $c(\xi )={\left(\text{ℂ}(\xi_{1},\xi ;\theta ),\ldots ,\text{ℂ}(\xi_{n},\xi );\theta \right)}^{T}$. We use an MLE of ***θ***, which is given by ${\text{arg max}}_{\theta }L(\theta )$, where $L(\theta )=\log p(\text{t}|\theta )=-(1/2)(\ln|\text{C}|+{\text{t}}^{T}{\text{C}}^{-1}\text{t}+n\ln(2\pi ))$ is the likelihood.
